# The Changing Face of Gastrointestinal Cancer Mortality: Trends and Divide by Age and Geography

**DOI:** 10.1245/s10434-026-19721-y

**Published:** 2026-04-27

**Authors:** Qaidar Alizai, Timothy M. Pawlik

**Affiliations:** Department of Surgery, The Ohio State University, Wexner Medical Center and James Comprehensive Cancer Center, Columbus, OH USA 43210

Risk-adjusted gastrointestinal (GI) cancer mortality varies by cancer type, age, time, and geography. Early-onset cancer (age < 50 years) is increasing for several cancers, particularly colorectal, breast, and thyroid cancers, and may reflect hereditary, lifestyle, and environmental factors. Early detection through symptom awareness, family history assessment, and timely screening or genetic testing may improve outcomes. This study examined U.S. age-adjusted GI cancer mortality trends by age group (age 20–49 vs. ≥ 50 years) and described cancer-specific temporal and geographic patterns.

## Methods

National Center for Health Statistics GI cancer mortality data were obtained from SEER*Stat, version 9.0.41.4. Age-adjusted mortality rates (2000 U.S. standard population) were analyzed from 1990 to 2023 for younger adults (age, 20–49 years) and older adults (age, ≥ 50 years) for all GI cancers combined and separately for esophageal, gastric, colorectal (CRC), liver/intrahepatic cholangiocarcinoma (ICC), and pancreatic cancers. The overall percentage change (PC) and the annual percentage change (APC) with 95% confidence intervals (CIs) were derived from SEER*Stat trend analyses. Temporal trends were displayed using age-stratified line graphs and county-level variation with percentile-based heat maps. Because de-identified public data were used, institutional review board approval and informed consent were not required.

## Results

Among younger adults, overall GI cancer mortality changed little from 1990 to 2023 (APC, − 0.1%; 95% CI − 0.2 to − 0.1%). Colorectal cancer was the only major subtype with worsening mortality, increasing from 1.7 per 100,000 in 2002–2004 to nearly 2.0 per 100,000 in 2019–2023. Mortality declined for esophageal cancer (APC, − 1.1%; 95% CI − 1.3 to − 0.8%), gastric cancer (APC, − 0.7%; 95% CI − 0.9 to − 0.6%), and pancreatic cancer (APC, − 0.5%; 95% CI − 0.6 to − 0.4%). Liver/ICC showed a mixed pattern, with an annual decline from 2002 to 2023, but an overall 10.4% increase from 1990 to 2023 (Table 1).

**Table 1:** Temporal trends in mortality rates of gastrointestinal cancers in the United States, stratified by age

Cancer type	Age < 50 years	Age < 50 years	Age ≥ 50 years	Age ≥ 50 years
	PC, (%)	APC, (%)/Year (95% CI)	PC, (%)	APC, (%)/Year (95% CI)
All GI cancers	− 2.7	− 0.1 (− 0.2 to − 0.1)	− 25.3	− 0.9 (− 0.9 to − 0.8)
Esophagus	− 25.1	− 1.1 (− 1.3 to − 0.8)	− 10.7	− 0.4 (− 0.6 to − 0.3)
Gastric	− 26.2	− 0.7 (− 0.9 to − 0.6)	− 59.9	− 2.8 (− 2.9 to − 2.7)
Colorectal	14.2	0.5 (0.3 to 0.6)	− 51.9	− 2.4 (− 2.5 to − 2.3)
Liver and ICC	10.4	− 0.5 (− 1.0 to 0.0)	91.3	2.1 (1.9 to 2.3)
Pancreas	− 20.4	− 0.5 (− 0.6 to − 0.4)	5.9	0.3 (0.2 to 0.3)

PC, percentage change; APC, annual percent change; CI, confidence interval; GI, gastrointestinal; ICC, intrahepatic cholangiocarcinoma

Among older adults, overall GI cancer mortality declined more substantially (APC, − 0.9%; 95% CI − 0.9 to − 0.8%). Colorectal cancer declined from approximately 79 per 100,000 in 2002–2004 to 48 per 100,000 in 2019–2023 (APC, − 2.4%; 95% CI − 2.5 to − 2.3%), and gastric cancer mortality also decreased markedly. In contrast, pancreatic cancer increased from about 55 to 60 per 100,000 and surpassed CRC in later years, whereas liver/ICC had the steepest increase (PC, 91.3%; APC, 2.1%; 95% CI 1.9 to 2.3%).

**Fig. 1:**
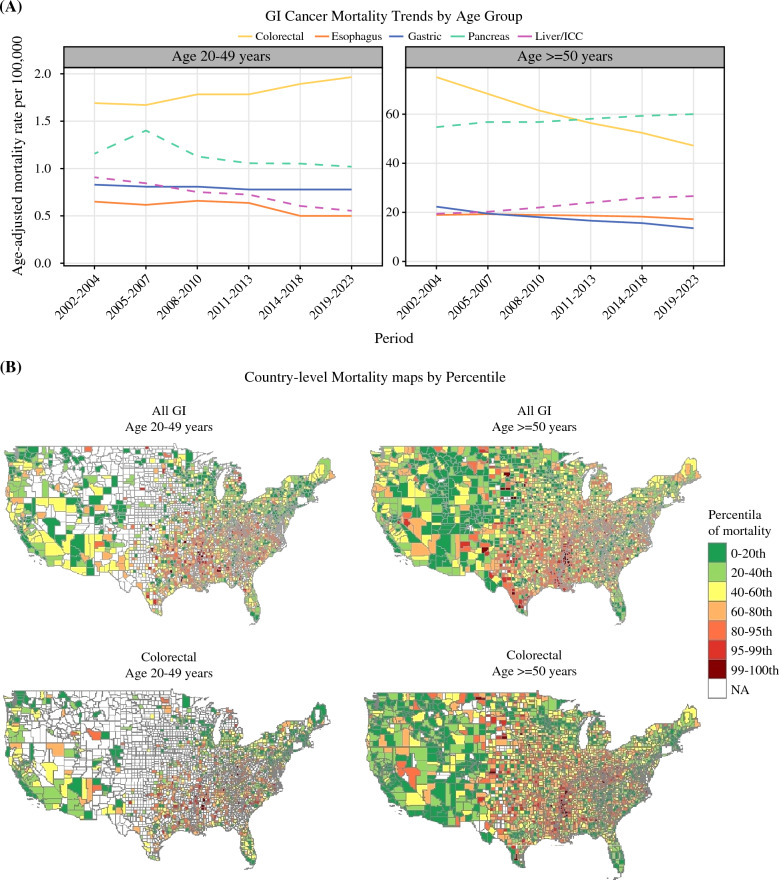
Temporal and geographic variations in mortality rates for young versus old-onset gastrointestinal cancers

County-level maps showed substantial geographic heterogeneity, with clearer clustering in older adults (Fig. 1). Higher-mortality counties were concentrated in the South, lower Mississippi Valley, Appalachia, and parts of the Southwest. Similar clustering was seen for CRC and pancreatic cancer. Esophageal cancer showed a stronger eastern/Appalachian pattern, whereas stomach cancer and liver/ICC were more concentrated in the Southwest, the Texas border region, the Gulf Coast, and the Southeast (Supplementary Fle 1).

## Discussion

Gastrointestinal cancer mortality trends differed sharply by age and subtype. Mortality improved little among adults younger than 50 years, largely because CRC mortality increased, whereas older adults experienced broader declines driven mainly by reductions in CRC and gastric cancer mortality. Rising younger-onset CRC mortality may reflect increasing disease burden, delayed recognition, and lower screening uptake, whereas favorable trends in older adults likely reflect screening, earlier detection, and treatment advances.^1–4^ These gains were partly offset by increasing pancreatic cancer mortality and a marked rise in liver/ICC mortality among older adults.

Regional clustering likely reflects differences in risk factors, socioeconomic conditions, prevention, timely diagnosis, and access to specialty cancer care.^5^ Reducing these disparities will require regionally targeted strategies, including strengthening prevention and risk-factor control, improving access to screening and diagnostic evaluation, and expanding equitable access to high-quality multidisciplinary cancer care.^5,6^

The current study was limited by its use of population-level mortality data without patient-level clinical information and descriptive geographic analyses. Nevertheless, these findings identify rising CRC mortality in younger adults and rising pancreatic and liver/ICC mortality in older adults as priority areas for intervention.

## Supplementary Information

Below is the link to the electronic supplementary material.

Supplementary file1 (DOCX 1696 KB)
