# Supplementary material for: The Changing Face of Gastrointestinal Cancer Mortality: Trends and Divide by Age and Geography
Source: Ann Surg Oncol. 2026 Apr 27;33(8):7418–21. doi: 10.1245/s10434-026-19721-y (PMC13337664; doi:10.1245/s10434-026-19721-y)

**The Changing Face of Gastrointestinal Cancer Mortality – Trends and Divide by Age and Geography**

**Supplementary Figure 1.** Mortality trends by geography and age for Esophageal and stomach cancers


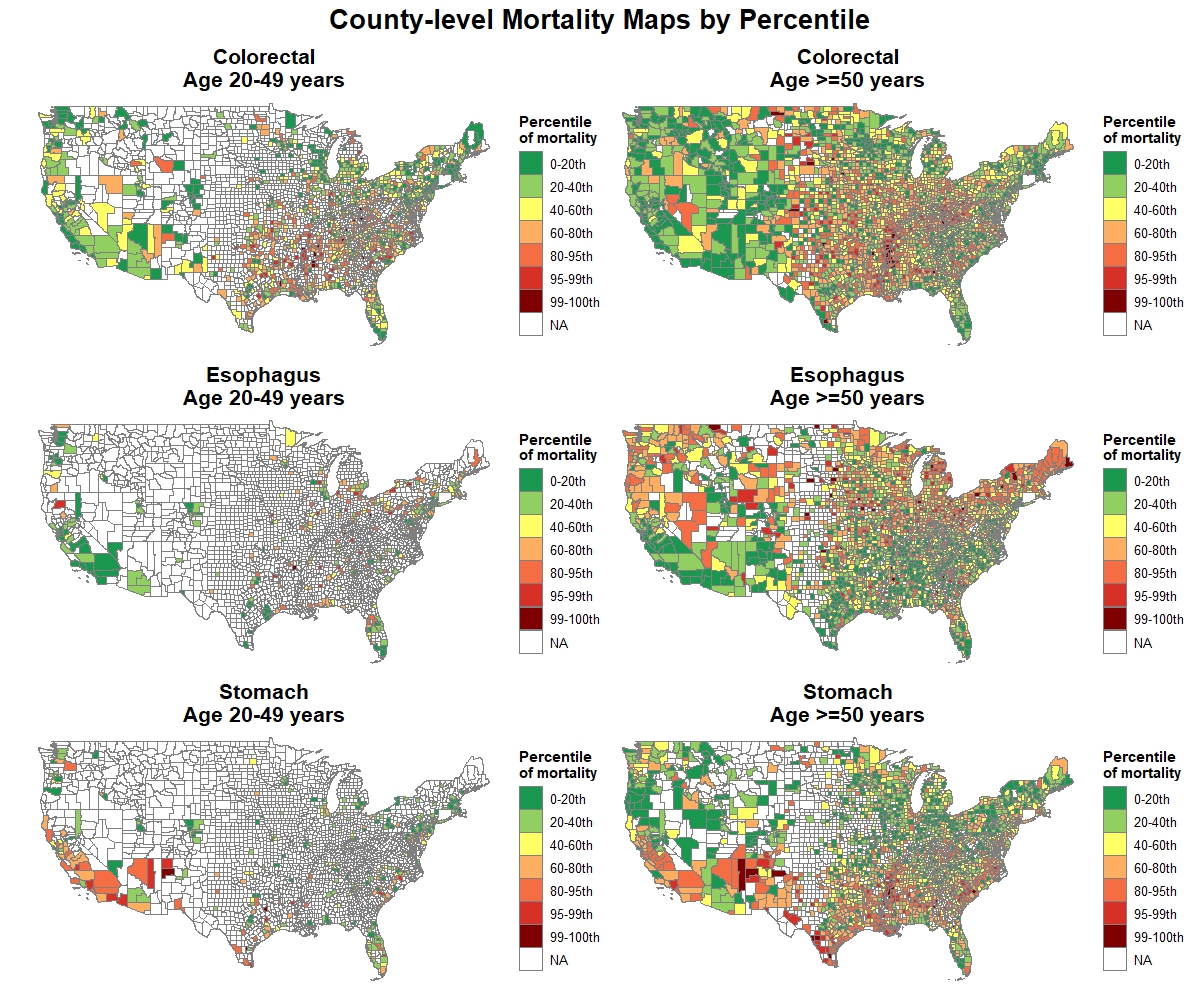


**Supplementary Figure 2.** Mortality trends by geography and age for Pancreatic and Liver/intrahepatic bile duct cancers.


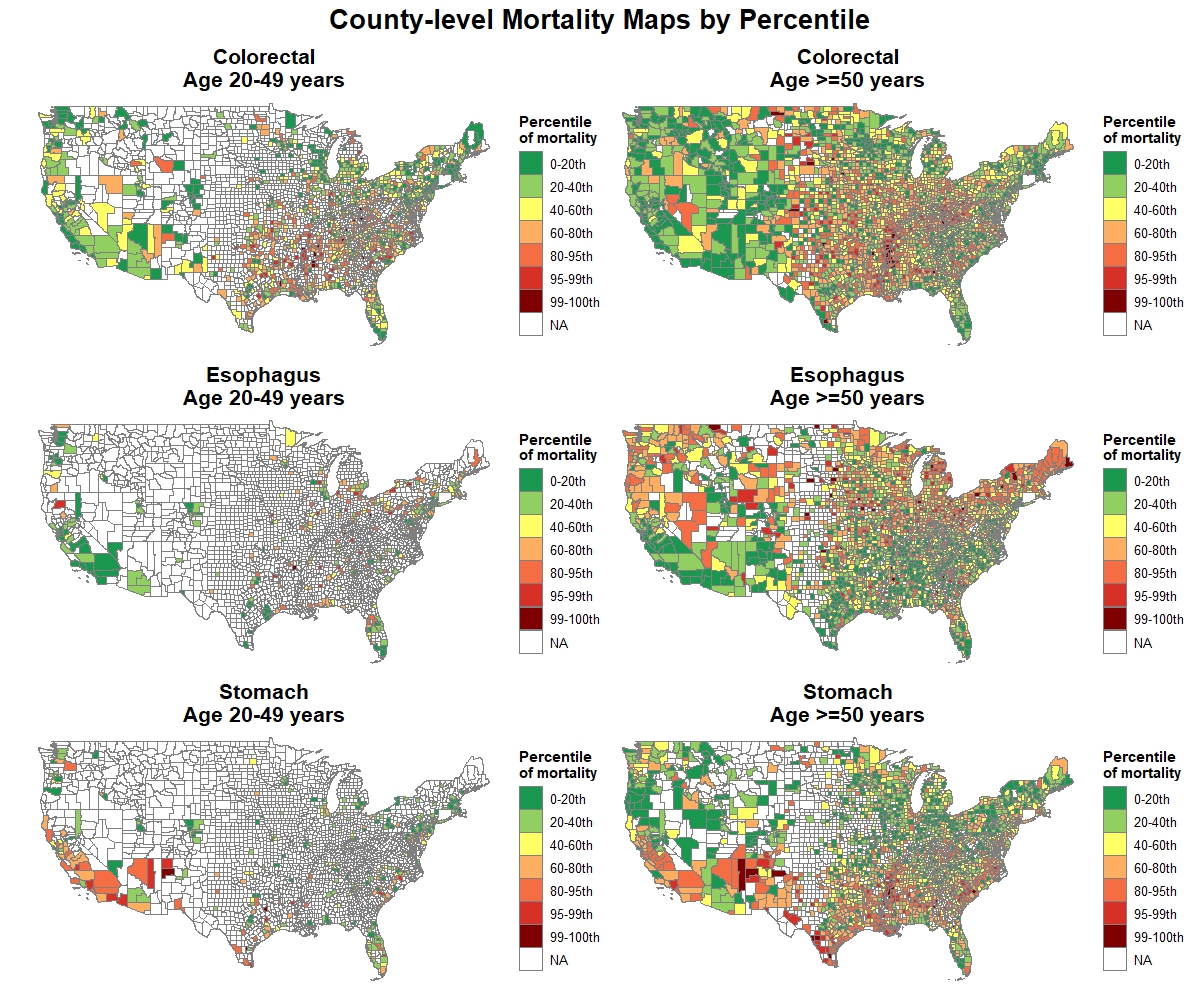

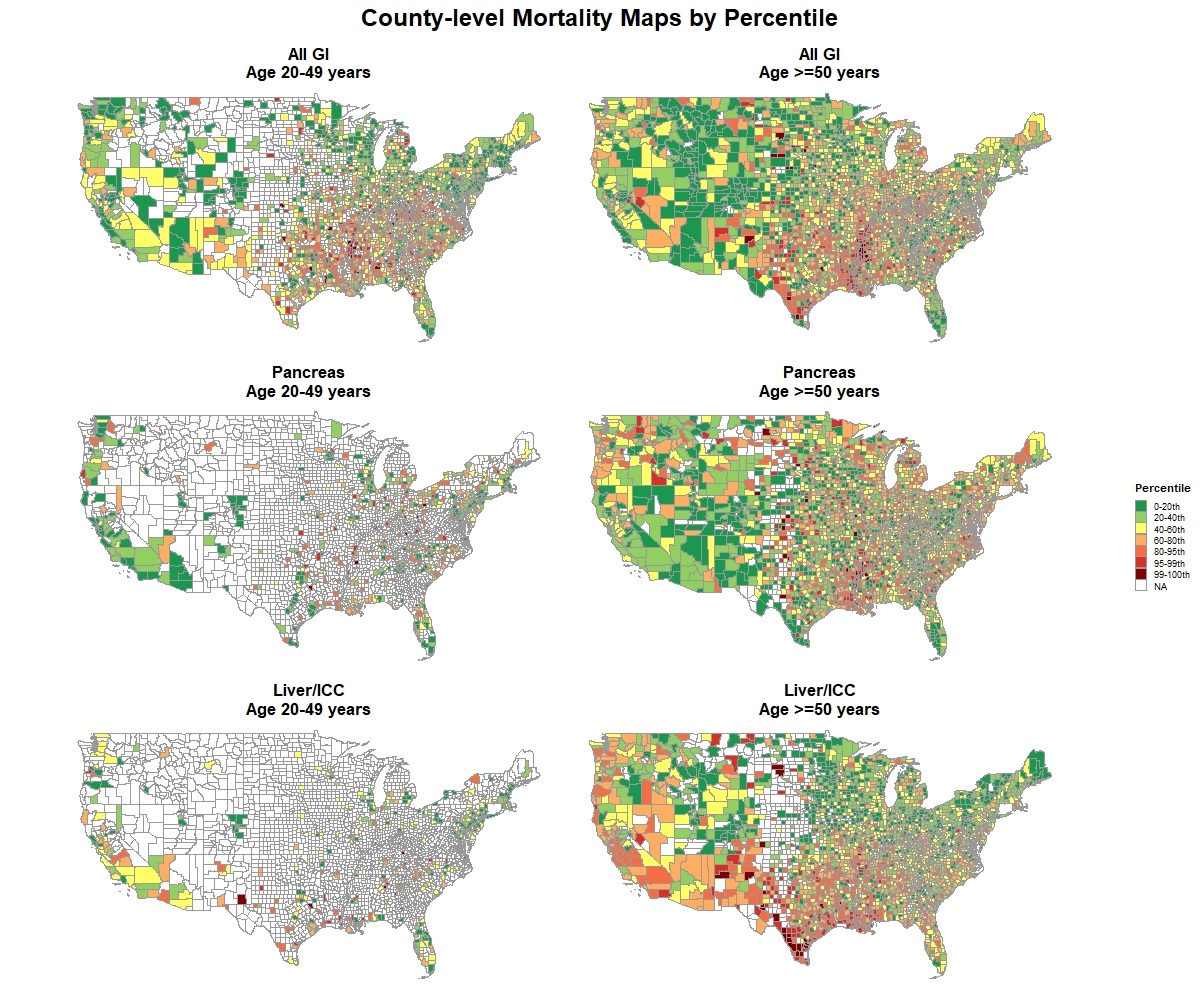

Supplement: Supplementary file 1 — Supplementary file1 (DOCX 1696 KB) [file 10434_2026_19721_MOESM1_ESM.docx]
